# Association between Serum Matrix Metalloproteinase- (MMP-) 3 Levels and Systemic Lupus Erythematosus: A Meta-analysis

**DOI:** 10.1155/2019/9796735

**Published:** 2019-07-18

**Authors:** Jiwon M. Lee, Andreas Kronbichler, Se Jin Park, Seong Heon Kim, Kyoung Hee Han, Hee Gyung Kang, Il Soo Ha, Hae Il Cheong, Ki Hwan Kim, Gaeun Kim, Dong Soo Kim, Hyun Wook Chae, Chul Ho Lee, Keum Hwa Lee, Jae Il Shin

**Affiliations:** ^1^Department of Pediatrics, Chungnam National University Hospital, Daejeon, Republic of Korea; ^2^Department of Internal Medicine IV (Nephrology and Hypertension), Medical University Innsbruck, Innsbruck, Austria; ^3^Department of Pediatrics, Ajou University Hospital, Ajou University School of Medicine, Suwon, Republic of Korea; ^4^Department of Pediatrics, Pusan National University Children's Hospital, Yangsan, Republic of Korea; ^5^Department of Pediatrics, Jeju National University School of Medicine, Jeju, Republic of Korea; ^6^Department of Pediatrics, Seoul National University Children's Hospital, Seoul, Republic of Korea; ^7^Department of Pediatrics, Incheon St. Mary's Hospital, The Catholic University of Korea, Seoul, Republic of Korea; ^8^Keimyung University College of Nursing, Daegu, Republic of Korea; ^9^Department of Pediatrics, Yonsei University College of Medicine, Seoul, Republic of Korea; ^10^Department of Pediatric Nephrology, Severance Children's Hospital, Seoul, Republic of Korea; ^11^Institute of Kidney Disease Research, Yonsei University College of Medicine, Seoul, Republic of Korea

## Abstract

**Introduction:**

Matrix metalloproteinase (MMP) is an emerging disease marker in rheumatic diseases. This is a meta-analysis aimed at systematically reviewing association between serum MMP-3 levels and systematic lupus erythematosus (SLE) activity, which sought to raise interest in MMP-3 as a putative biomarker.

**Methods:**

We conducted a meta-analysis of serum MMP-3 levels in patients with SLE and controls. We performed a PubMed search, EMBASE search, and forward search of the retrieved articles published until Oct. 1, 2018. In addition to this, we included data from a case-control study on a national pediatric SLE cohort, in which serum MMP-3 levels were measured in 11 SLE patients and 9 controls (unpublished). Subgroup analyses based on gender and disease activity were performed.

**Results:**

A total of 662 cases and 771 controls including 651 patients and 762 controls from 11 publications were studied. We observed significantly higher MMP-3 levels in SLE patients compared to healthy controls (*P* < 0.001, Hedges' *g*: 2.104, 95% CI 1.426-2.782). In subgroup analyses, we found a significant elevation of MMP-3 in the patients with nephritis compared to those without (*P* = 0.006, Hedges' *g*: 0.611, 95% CI 0.611-1.704). This finding was consistent between patients with persistent proteinuria and those without (*P* = 0.023, Hedges' *g*: 1.535, 95% CI 0.207-2.862). Meta-analysis showed no association between MMP-3 levels and gender or anti-double strand DNA antibody titer.

**Conclusions:**

Our meta-analysis demonstrated significantly higher MMP-3 levels in SLE patients than in controls and in patients with renal involvement than in those without.

## 1. Introduction

Systemic lupus erythematosus (SLE) is a multisystemic autoimmune disease [1]. Although the pathogenesis of SLE remains yet to be elucidated, studies have reported its association with dysregulation of matrix metalloproteinases (MMPs) [2, 3]. MMPs, a family of enzymes, were discovered for the ability to degrade extracellular matrix (ECM) and basement membrane components [4]. Since they have important roles in wound healing through processes implicated in tissue remodeling [4, 5], an imbalance between MMPs and their endogenous inhibitors, such as tissue inhibitors of metalloproteinases (TIMPs), may lead to tissue destruction and associated inflammatory diseases [4, 5].

A body of literature investigated MMP as a potential biomarker in various rheumatic diseases, namely, rheumatoid arthritis, Kawasaki disease, giant cell arteritis, Takayasu arteritis, and anti-neutrophil cytoplasmic antibody (ANCA) -associatedvasculitis [6–10]. In addition, our group has previously studied the expression profiles of all known MMPs and TIMPs in children with IgA vasculitis (former Henoch-Schönlein purpura (HSP)) [11]. Increased levels of MMPs and TIMPs in children with IgA vasculitis were observed [11]. In patients with SLE, MMPs including MMP-2, 3, 9, and 13 are proposed to correlate with SLE activity [2, 3, 12, 13]. However, conflicting data on serum MMP-3 levels and its correlation with SLE [2, 3, 12] prompted us to further investigate its role.

We performed this meta-analysis to review serum MMP-3 levels in patients with SLE compared to those in healthy controls and determine the correlation of MMP-3 levels with disease activity of SLE.

## 2. Methods

### 2.1. Search Strategy and Data Extraction

We performed a PubMed, EMBASE, and Google Scholar search to identify eligible articles. Furthermore, a forward search of the retrieved articles was performed, and “Google Scholar” was assessed to screen for nonindexed publications. The last search in EMBASE and PubMed was performed on Oct. 1, 2018. The search terms included the following: systemic lupus erythematosus OR “SLE” OR “lupus” OR “lupus nephritis” AND matrix metalloproteinase 3 OR “matrix metalloproteinase-3” OR “MMP 3” OR “MMP-3” OR “Stromelysin 1” OR “Stromelysin-1”. The detailed search strategy is as follows.

PubMed and MEDLINE search strategy (last search performed on Oct. 1, 2018):

#1 “systemic lupus erythematosus” [All Fields] or [Mesh]
#2 “lupus nephritis” [All Fields]
#3 “lupus” [All Fields]
#4 #1 OR #2 OR #3
#5 “matrix metalloproteinase 3” [All Fields] or [Mesh]
#6 “matrix metalloprotease-3” [All Fields]
#7 “MMP 3” [All Fields] OR “MMP-3” [All Fields]
#8 “Stromelysin 1” [All Fields] OR “Stromelysin-1” [All Fields]
#9 #5 OR #6 OR #7 OR #8
#10 #4 AND #9

**Figure 1:**
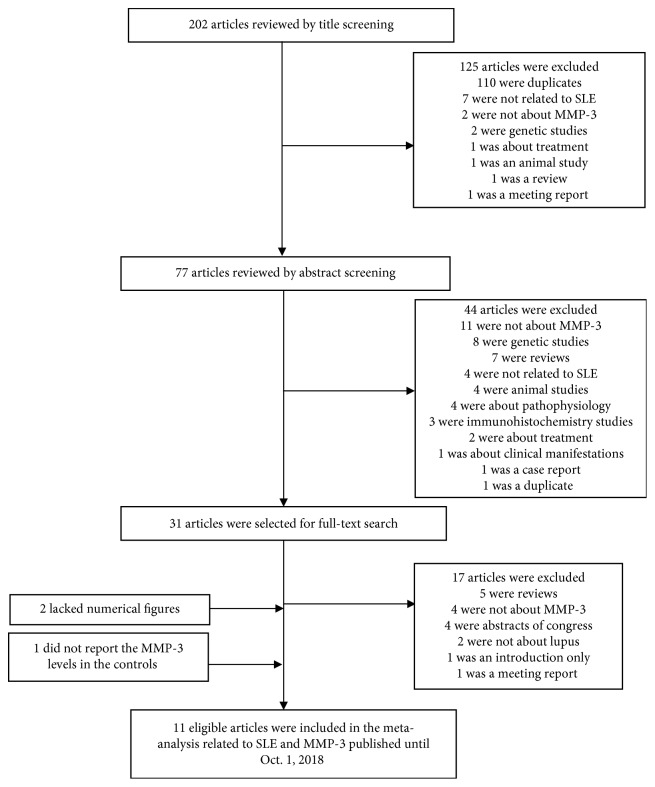
Flow diagram of search strategy.

We examined and screened the articles firstly by titles, followed by abstracts, and eventually by assessing and reading the respective full texts. The detailed process of reviewing the articles is presented in Figure 1.

### 2.2. Eligibility Criteria

We included cross-sectional or longitudinal studies which measured MMP-3 levels in the sera of patients with SLE and compared them with controls. We excluded studies that measured MMP-3 in the joint fluid or kidney tissues. Animal studies were also excluded. The decision to include or exclude was made independently by two authors (Lee JM and Shin JI), and any disagreements were settled by discussion.

### 2.3. Quality Assessment

The meta-analysis followed the Preferred Reporting Items for Systematic Reviews and Meta-Analysis (PRISMA) statement (Supplementary Table S1). We used the Newcastle-Ottawa Scale (NOS) [14] to score the quality of the studies, recommended by the Cochrane Collaboration [14]. The scoring was performed independently by two researchers (Shin JI and Lee JM). The NOS ranges from 0 to 9 stars; a study can be awarded a maximum of one star for each numbered item within the Selection and Exposure categories. A maximum of two stars can be given for comparability. If more than 6 stars were given, the study is assumed to have a high quality (Supplementary Table S2).

### 2.4. Unpublished Data from Pediatric SLE Cohort

In order to reinforce the power of the meta-analysis, we included our data from a case-control study, which we performed earlier on the national pediatric SLE cohort (KPS) (data not published). We were able to quantify serum MMP-3 levels in 11 children with SLE and 9 healthy controls. Detailed information with regard to this cross-sectional study is provided as Supplementary Materials and in Supplementary Table S3.

### 2.5. Statistical Analysis and Evaluation of Heterogeneity and Publication Bias

We calculated Hedges' *g*, and corresponding 95% confidence intervals (CIs) were used to compare serum MMP-3 levels. All meta-analyses were performed using random and fixed effects models, but only random effects models were used because true differences among the studies were expected due to heterogeneity.

We assessed the heterogeneity of the studies by using the Cochran *Q* test, and a *P* value of < 0.05 was considered significant. The inconsistency across the studies was also measured by *I* ^2^ metric, as a measure of the percentage of total variation across the studies because of heterogeneity. *I* ^2^ values of <25, 25-75, and >75% were considered to represent low, moderate, and high levels of heterogeneity, respectively. Publication bias of each article was estimated by inspecting a funnel plot and using the Egger test. All analyses were conducted using Comprehensive Meta-Analysis v.2.0 (Biostat, Englewood, NJ, USA).

## 3. Results

### 3.1. Study Selection and Characteristics

We were able to identify 202 articles using electronic and manual researches. After reviewing titles and abstracts, 31 studies were selected for full-text reading. Of them, 17 were excluded due to duplicates, irrelevance, or inappropriateness. Of the remaining 14 studies, 3 were excluded (2 lacked numerical figures, and 1 did not report MMP-3 levels in controls) to finally include 11 eligible articles (Figure 1) [2, 3, 12, 15–22]. Here, we included results from a national pediatric SLE cohort (KPS) involving 11 SLE and 9 healthy controls.

The respective characteristics of included studies are summarized in Table 1. The PRISMA checklist for meta-analyses is shown in Supplementary Table S1. Study quality assessed by using the Newcastle-Ottawa scale (NOS) scored 6 in two studies, 7 in three studies, and 8 in four studies (range: 1 (very poor) to 9 (very high); Supplementary Table S2).

### 3.2. Meta-analysis of MMP-3 Levels in SLE Patients Compared to Controls

**Figure 2:**
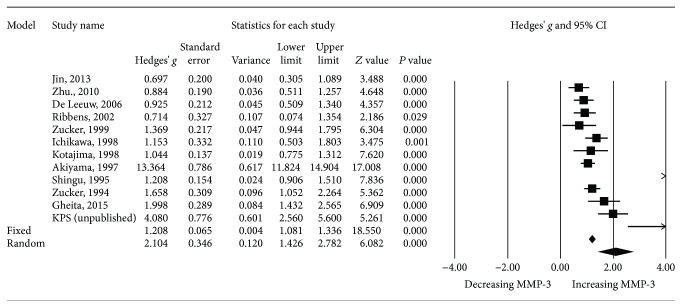
Forest plot of random effects meta-analysis of MMP-3 levels in SLE patients compared with healthy controls.

**Figure 3:**
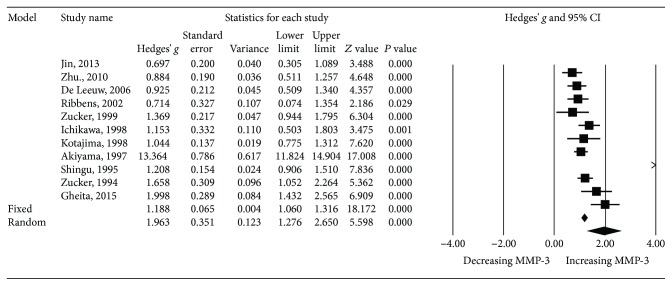
Forest plot of random effects meta-analysis of MMP-3 levels in SLE patients compared with healthy controls (excluding pediatric data from KPS).

A meta-analysis on SLE patients and healthy controls was performed. Extracting data from 12 studies (11 published articles and KPS data), there were 662 patients with SLE and 771 controls. The results revealed that MMP-3 levels were significantly higher in the SLE group than in the control group (*P* < 0.001, Hedges' *g*: 2.104, 95% CI 1.426-2.782) (Table 2 and Figure 2). We then performed the same analysis excluding our KPS data to confirm that the results were not affected by including pediatric data. The results consistently showed that MMP-3 levels were significantly higher in SLE patients than in controls (*P* = 0.001, Hedges' *g*: 1.963, 95% CI 1.276-2.650) (Table 2 and Figure 3).

### 3.3. Meta-analysis of MMP-3 Levels in Subgroups by Gender

**Figure 4:**
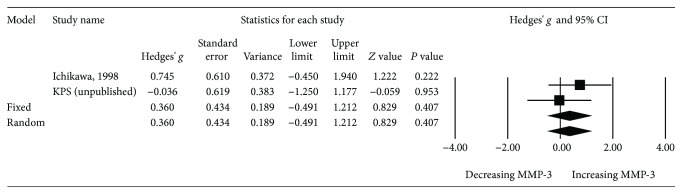
Forest plot of random effects meta-analysis of MMP-3 levels in SLE patients; male vs. female.

In subgroup analyses, we firstly compared serum MMP-3 levels in male vs. female SLE patients. Data were extracted from two studies; Ichikawa et al. [18] and KPS (unpublished). The results revealed no significant difference (*P* = 0.407, Hedges' *g*: 0.360, 95% CI -0.491-1.212) (Figure 4).

### 3.4. Meta-analysis of MMP-3 Levels in SLE Patients with Renal Involvement and Those Without

**Figure 5:**
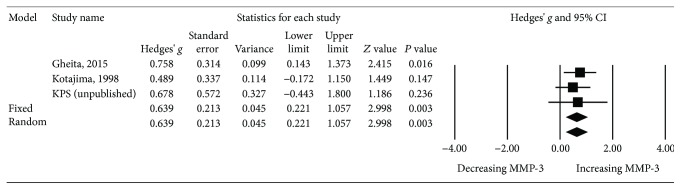
Forest plot of random effects meta-analysis of MMP-3 levels in SLE patients; with vs. without nephritis.

We compared serum MMP-3 levels in SLE patients with active nephritis (*n* = 53) and those without (*n* = 39). Data were extracted from three studies. Kotajima et al. [2] defined active nephritis according to the SLEDAI score, while Gheita et al. [22] and KPS (unpublished) defined it as biopsy-proven nephritis. The meta-analysis showed that MMP-3 levels were significantly higher in the lupus nephritis group than in the nonnephritis group (*P* = 0.003, Hedges' *g*: 0.639, 95% CI 0.221-1.057) (Table 2 and Figure 5).

**Figure 6:**
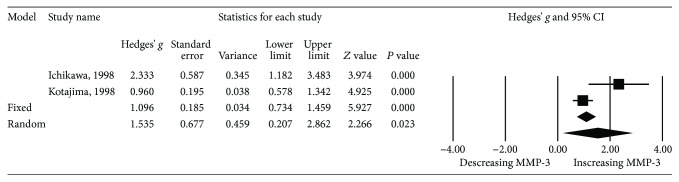
Forest plot of random effects meta-analysis of MMP-3 levels in SLE patients; with vs. without proteinuria.

In addition, subgroup meta-analysis involving two studies [2, 18] was performed on patients with proteinuria (*n* = 57) and those without (*n* = 82). Proteinuria was defined as >0.5 gm/24 hours according to the SLEDAI score. The results revealed that serum MMP-3 levels were significantly higher in patients with overt proteinuria than in those without (*P* = 0.028, Hedges' *g*: 1.583, 95% CI 0.167-3.000) (Table 2 and Figure 6).

### 3.5. Meta-analysis of MMP-3 Levels of SLE Patients with Positive Anti-dsDNA Titer

**Figure 7:**
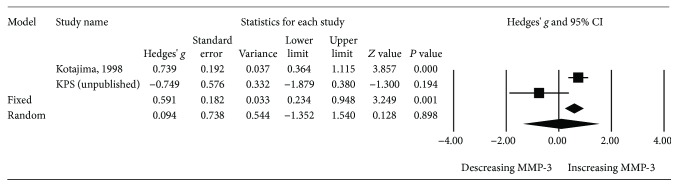
Forest plot of random effects meta-analysis of MMP-3 levels in SLE patients; with vs. without increased anti-dsDNA titer.

Further meta-analyses were conducted on SLE patients in subgroups based on abnormal anti-double strand DNA antibody (anti-dsDNA Ab) titer at the time of sample collection. The results involving two studies—Kotajima et al. [2] and KPS (unpublished)—demonstrated no significant difference in MMP-3 levels between SLE patients with abnormally increased anti-dsDNA Ab titer and those without (*P* = 0.898, Hedges' *g*: 0.094, 95% CI -1.325-1.540) (Table 2 and Figure 7).

### 3.6. Assessment of Heterogeneity and Publication Bias

**Figure 8:**
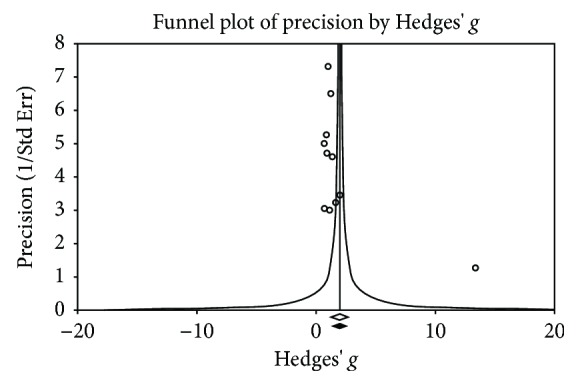
Funnel plot of standard error in meta-analysis of MMP-3 levels in SLE patients compared with healthy controls.

We assessed statistical heterogeneity between the included studies (Table 2). In the meta-analysis of serum MMP-3 levels comparing SLE patients with healthy controls and subgroup analysis of proteinuria, the *I* ^2^ test showed a value > 50%, indicating substantial heterogeneity. Random effects models were used for meta-analyses. Although the funnel plot showed symmetry (Figure 8), Egger's regression analysis indicated possibility of publication bias (Table 2).

## 4. Discussion

Due to a remitting-relapsing disease course of most patients with SLE, biomarkers reflecting disease activity are desirable. One of the candidate biomarkers is the MMP family. MMP-3, also known as Stromelysin-1, degrades tissue proteins including collagen types II, III, IV, IX, and X, proteoglycans, fibronectin, laminin, and elastin [12]. It can also activate other MMPs, such as MMP-9, which is suggested to be involved in the pathogenesis of SLE [13]. A recently published meta-analysis involving 12 studies, however, showed that circulating MMP-9 levels did not differ between SLE patients and healthy controls [23].

In this meta-analysis, serum MMP-3 levels were reviewed in 662 SLE patients and 771 controls. There were 621 patients and 762 controls extracted from 11 publications and 11 patients and 9 controls from a pediatric lupus cohort, KPS. The results showed firstly that serum MMP-3 levels were significantly higher in patients with SLE than in healthy controls and secondly that serum MMP-3 levels were significantly elevated in patients with renal involvement than in those without, both for active lupus nephritis and persistent proteinuria. Previous studies suggested a correlation of serum MMP-3 levels and hematologic indices, such as white blood cells (WBC) and platelet counts [22]. However, in our meta-analysis, subgroup comparisons were available only for renal manifestations, sex, and serum anti-dsDNA antibody titer due to paucity of quantifiable data. Subgroup comparison by sex and serum anti-dsDNA antibody titer showed no significant difference in the serum MMP-3 levels.

With regard to MMP-3, several studies have reported elevation of circulatory MMP-3 levels in SLE patients [2, 3, 12, 17, 22]. Our meta-analysis results were in agreement with these studies. However, the correlation of serum MMP-3 elevation and disease activity of SLE had been inconsistent [2, 3, 12, 22]. Precisely, Kotajima et al. reported that increased levels of serum MMP-3 in SLE are related to clinical features relevant to lupus nephritis [2]. They found that serum MMP-3 levels were significantly higher in SLE patients with active clinical presentation such as persistent proteinuria, malar rash, and laboratory parameters, such as cellular casts, anti-dsDNA antibodies, decreased complement C3 and C4 levels, circulating immune complexes, and hypoalbuminemia [2]. Similarly, Gheita et al. found that serum levels of MMP-3 correlated with the systemic lupus erythematosus disease activity index (SLEDAI) and Systemic Lupus International Collaborating Clinics/damage index (SLICC/DI) scores [22]. However, Zucker et al. found an increase in serum concentrations of MMP-3 in SLE but reported no correlation with disease activity [12]. Moreover, Zhu et al. [3] reported that serum MMP-2, MMP-3, and MMP-13 levels in SLE patients were significantly higher than those in controls but found no overall correlation between serum levels of the three MMPs and disease activity scores. Our data supported the relationship between serum MMP-3 levels and renal involvement of SLE, implicating its correlation with disease activity. With regard to renal involvement, a few studies investigated its association with serum MMP levels. In a study by Gheita et al., the serum MMP-3 levels correlated with class of lupus nephritis, showing the highest levels in patients with class IV nephritis [22]. Thiyagarajan et al. speculated in an animal study that MMPs may represent some component of membrane disintegration in progressive nephritis [24]. Our results and previous works suggested that serum MMP-3 levels may reflect the presence and possibly histological severity of lupus nephritis in patients with SLE.

There are several limitations in this study. First, the mean values of MMP-3 serum levels in SLE patients were relatively high in those studies published in more remote years (before the year 2000) and significantly lower in those studies performed after 2000. We speculate that different ELISA kits may have made a general comparability of results impossible. This issue led to different nonreproducible results in the past (biomarker biology), but we have only included studies with respective control cohorts and observed similar regulation in most studies. Still, this is a major limitation in this study. Second, this meta-analysis had small sample sizes, lowering the power of the study. In those meta-analyses involving two studies, the conclusions drawn may be subject to bias because they are affected by the small sample size of clinical studies. In order to alleviate this, we used a random effects model in this study. However, we speculate that such a limitation should raise attention and subsequently increase publications in this subject. This is one of the reasons for performing this work. Third, the data included in this meta-analysis are extracted from heterogeneous groups. The patients had different demographics, such as age, sex, and ethnicity, and varying clinical manifestations which may have affected the results. In particular, this meta-analysis included data from one pediatric cohort (KPS) and 11 studies on adult patients which may have increased heterogeneity of the data. Lastly, there remains a possibility of existing literature that was not accessible and the presence of publication bias.

Although the results require cautious interpretation, we speculate that this meta-analysis may provide some evidence-based results regarding a controversial issue, based on current publications. In the future, meta-analysis using individual patient data and propensity scoring would make a more powerful study.

Firstly, the results of the present study revealed that serum MMP-3 levels were significantly elevated in SLE patients, which is in accordance with previous reports [2, 3, 12, 22]. Secondly, the results showed that MMP-3 was significantly elevated in patients with renal involvement, both in histologically proven lupus nephritis and mere proteinuria. Although the correlation of MMP-3 and lupus activity requires further verification, it is yet tempting to speculate that elevated MMP-3 at initial diagnosis of SLE may require more close follow-ups.

## 5. Conclusions

The present meta-analysis showed that serum MMP-3 levels were significantly higher in patients with SLE than in controls and in patients with renal involvement than in those without. Although our meta-analysis suggested that MMP-3 likely correlate with disease activity, further studies in a larger scale are warranted to elucidate the role of MMP-3 as a putative biomarker of SLE.

## Figures and Tables

**Table 1:** Characteristics of all studies included in the meta-analysis.

Author (year)	Study groups	*N*	Sex	Age	Subgroup analysis within SLE	MMP-3 in SLE	MMP-3 in HC	ELISA kit used
			M/F	Mean (SD or range)		Mean (SD or range)	Mean (SD or range)	
Jin et al. (2013) [15]	SLE	31	0/31	48.7 (12.7)	—	25.2 (23.4)	10.2 (4.4)	Quantikine; R&D Systems Inc., UK
	HC	150	0/150	45.8 (11.3)				

Zhu et al. (2015)	SLE	60	3/57	33.3 (10.8)	—	21.4 (7.3)	14.4 (8.4)	Quantikine; R&D Systems Inc., UK
	HC	60	3/57	33.4 (10.9)				

De Leeuw et al. (2006) [16]	SLE	72	9/63	41.0 (12.0)	—	19 (14.1)	8.0 (4.4)	BioSource, Europe S.A., Belgium
	HC	36	3/33	41.0 (12.0)				

Ribbens et al. (2002) [17]	Cutaneous lupus	7	0/7	34 (15-57)	Cutaneous lupus	11.5 (4.4) (female data)	9.2 (2.8)	BioSource, Europe S.A., Belgium
	Renal lupus	7	2/5	30 (15-54)	Renal lupus			
	HC	96	50/46	44 (25-64)				

Zucker et al. (1999) [12]	SLE	73	N/A	N/A	—	416.0 (252.0)	125.0 (93.0)	In-house method using human Ab (Mac078, Celltech)
	HC	39	N/A	N/A				

Ichikawa et al. (1998) [18]	SLE	21	3/18	38.2 (13.5)	—	239.1 (199.6)	63 (64.1)	Fuji Chemical Industries Ltd., Toyama, Japan
	HC	20	0/20	56.8 (4.6)				

Kotajima et al. (1998)	SLE	124	8/116	N/A	With or without dsDNA	193.0 (171.5)	61.8 (33.9)	In-house method using human Ab (NB1RGB)
	HC	117	67/50	N/A				

Akiyama et al. (1997) [19]	SLE	13	5/7	35.6 (3.5)	—	155.7 (23.4)	61.7 (3.15)	Fuji Chemical Industries Ltd., Toyama, Japan
	HC	154	N/A	N/A				

Shingu et al. (1995) [20]	SLE	67	6/61	46.5 (15.6)	—	117.3 (107.4)	42.1 (29.2)	Fuji Chemical Industries Ltd., Toyama, Japan
	HC	170	50/120	N/A				

Zucker et al. (1994) [21]	SLE	17	3/14	36.2 (N/A)	—	258.4 (124.3)	50.0 (124.3)	In-house method using human Ab (Mac078, Celltech)
	HC	53	30/23	42.0 (N/A)				

Gheita et al. (2015) [22]	SLE	42	0/42	33.2 (11.6)	With or without nephritis	80.9 (45.8)	10.01 (2.6)	N/A
	HC	30	0/30	N/A				

KPS study (unpublished)	SLE	11	3/8	14.5 (11.8-18)	With vs. without nephritis	195.3 (50.3)	26.4 (19.3)	Ab Frontier, Seoul, Korea
	HC	9	2/7	12.2 (10.0-15.0)	With vs. without dsDNA			

Abbreviations used: dsDNA: double-stranded DNA antibodies; F: female; HC: healthy controls; M: male; MMP-3: matrix metalloproteinase-3; *N*: number; N/A: not available; SD: standard deviation; SLE: systemic lupus erythematosus. MMP-3 levels were all measured in ng/mL.

**Table 2:** Summary of the results of meta-analysis.

Group-wise	No. of studies	No. of subjects		Meta-analysis				Heterogeneity			Egger's bias
				Hedges' *g*	95% CI		*P* value	*I* ^2^ (%)	Tau^2^	*P* value	*P* value
*Total*											
SLE vs. HC (including KPS data)	12	SLE 662	HC 771	2.104	1.426	2.782	<0.001	96.046	1.308	<0.001	0.02
SLE vs. HC (excluding KPS data)	11	SLE 651	HC 762	1.963	1.276	2.650	<0.001	96.217	1.256	<0.001	0.02
*Sex*											
Male vs. female SLE	2	Male 6	Female 26	0.360	-0.491	1.212	0.407	0.000	0.000	0.369	—
*Renal manifestation*											
SLE with vs. without nephritis	3	With 53	Without 39	0.639	0.221	1.057	0.003	0.000	0.000	0.841	—
SLE with vs. without proteinuria	2	With 57	Without 82	1.535	0.207	2.862	0.023	79.694	0.751	0.023	—
*Disease activity*											
SLE (+) vs. (-) anti-dsDNA	2	(+) 57	(-) 82	0.094	-1.352	1.540	0.898	83.360	0.924	0.014	—

Abbreviations used: dsDNA: double-stranded DNA antibodies; HC: healthy controls; SLE: systemic lupus erythematosus. *P* values were all two-tailed.

## Data Availability

The raw data supporting this meta-analysis are from previously reported studies and datasets, which have been cited and included as supplementary material. The processed data are included within the article and Supplementary Materials. The full processed data in detail are also available from the corresponding author upon request.

## Supplementary Materials

Supplementary Materials Supplementary Table S1: PRISMA 2009 Checklist. Supplementary Table S2: the Newcastle-Ottawa Scale (NOS). Supplementary Table S3: comparison of investigated biomarkers in pediatric SLE patients and controls (KPS data). Supplementary Material on pediatric SLE (KPS) data.
