# Corrigendum to “Association between Serum Matrix Metalloproteinase- (MMP-) 3 Levels and Systemic Lupus Erythematosus: A Meta-Analysis”

**DOI:** 10.1155/2020/5943216

**Published:** 2020-01-23

**Authors:** Jiwon M. Lee, Andreas Kronbichler, Se Jin Park, Seong Heon Kim, Kyoung Hee Han, Hee Gyung Kang, Il Soo Ha, Hae Il Cheong, Ki Hwan Kim, Gaeun Kim, Dong Soo Kim, Hyun Wook Chae, Chul Ho Lee, Keum Hwa Lee, Jae Il Shin

**Affiliations:** ^1^Department of Pediatrics, Chungnam National University Hospital, Daejeon, Republic of Korea; ^2^Department of Internal Medicine IV (Nephrology and Hypertension), Medical University Innsbruck, Innsbruck, Austria; ^3^Department of Pediatrics, Ajou University Hospital, Ajou University School of Medicine, Suwon, Republic of Korea; ^4^Department of Pediatrics, Pusan National University Children's Hospital, Yangsan, Republic of Korea; ^5^Department of Pediatrics, Jeju National University School of Medicine, Jeju, Republic of Korea; ^6^Department of Pediatrics, Seoul National University Children's Hospital, Seoul, Republic of Korea; ^7^Department of Pediatrics, Incheon St. Mary's Hospital, The Catholic University of Korea, Seoul, Republic of Korea; ^8^Keimyung University College of Nursing, Daegu, Republic of Korea; ^9^Department of Pediatrics, Yonsei University College of Medicine, Seoul, Republic of Korea; ^10^Department of Pediatric Nephrology, Severance Children's Hospital, Seoul, Republic of Korea; ^11^Institute of Kidney Disease Research, Yonsei University College of Medicine, Seoul, Republic of Korea

In the article titled “Corrigendum to “Association between Serum Matrix Metalloproteinase- (MMP-) 3 Levels and Systemic Lupus Erythematosus: A Meta-analysis”” [1], an acknowledgement should be added as follows:

The experiments for MMP-3 were supported by a grant from the Korean Society of Pediatric Nephrology.
