# Supplementary material for: Association between Serum Matrix Metalloproteinase- (MMP-) 3 Levels and Systemic Lupus Erythematosus: A Meta-analysis
Source: Dis Markers. 2019 Jul 18;2019:9796735. doi: 10.1155/2019/9796735 (PMC6668546; doi:10.1155/2019/9796735)
Supplement: Supplementary Materials — Supplementary Table S1: PRISMA 2009 Checklist. Supplementary Table S2: the Newcastle-Ottawa Scale (NOS). Supplementary Table S3: comparison of investigated biomarkers in pediatric SLE patients and controls (KPS data). Supplementary Material on pediatric SLE (KPS) data. [file 9796735.f1.docx]

**Association between serum matrix-metalloproteinase (MMP)-3 levels and systemic lupus erythematosus**

**: a meta-analysis**

Jiwon M. Lee^1,^*, Andreas Kronbichler^2,^*, Se Jin Park^3,^*, Seong Heon Kim^4,^*, Kyoung Hee Han^5^,

Hee Gyung Kang^6^, Il Soo Ha^6^, Hae Il Cheong^6^, Ki Hwan Kim^7^, Gaeun Kim^8^,

Dong Soo Kim^9^, Hyun Wook Chae^9^, Chul Ho Lee^9^, Keum Hwa Lee^9^ and Jae Il Shin^9,10,11^

^1^Department of Pediatrics, Chungnam National University Hospital, Daejeon, Korea

^2^Department of Internal Medicine IV (Nephrology and Hypertension), Medical University Innsbruck, Innsbruck, Austria

^3^Department of Pediatrics, Ajou University Hospital, Ajou University School of Medicine, Suwon, Korea

^4^Department of Pediatrics, Pusan National University Children’s Hospital, Yangsan, Korea

^5^Department of Pediatrics, Jeju National University School of Medicine, Jeju, Korea

^6^Department of Pediatrics, Seoul National University Children’s Hospital, Seoul, Korea

^7^Department of Pediatrics, Incheon St.Mary’s Hospital, the Catholic University of Korea, Seoul, Korea

^8^Keimyung University College of Nursing, Daegu, Korea

^9^Department of Pediatrics, Yonsei University College of Medicine, Seoul, Korea

^10^Department of Pediatric Nephrology, Severance Children’s Hospital, Seoul, Korea

^11^Institute of Kidney Disease Research, Yonsei University College of Medicine, Seoul, Korea

**Supplementary Table S1.** PRISMA 2009 Checklist

**Supplementary Table S2.** The Newcastle-Ottawa Scale (NOS)

**Supplementary Table S3.** Comparison of investigated biomarkers in pediatric SLE patients and controls (KPS data).

**Supplementary Material on pediatric SLE (KPS) data**

**Supplementary Table 1.** PRISMA 2009 Checklist

| **Section/topic** | **#** | **Checklist item** | **Reported on page #** |
| --- | --- | --- | --- |
| **TITLE** | | |  |
| Title | 1 | Identify the report as a systematic review, meta-analysis, or both. | 1 |
| **ABSTRACT** | | |  |
| Structured summary | 2 | Provide a structured summary including, as applicable: background; objectives; data sources; study eligibility criteria, participants, and interventions; study appraisal and synthesis methods; results; limitations; conclusions and implications of key findings; systematic review registration number. | 3 |
| **INTRODUCTION** | | |  |
| Rationale | 3 | Describe the rationale for the review in the context of what is already known. | 4 |
| Objectives | 4 | Provide an explicit statement of questions being addressed with reference to participants, interventions, comparisons, outcomes, and study design (PICOS). | 4 |
| **METHODS** | | |  |
| Protocol and registration | 5 | Indicate if a review protocol exists, if and where it can be accessed (e.g., Web address), and, if available, provide registration information including registration number. | 5 |
| Eligibility criteria | 6 | Specify study characteristics (e.g., PICOS, length of follow-up) and report characteristics (e.g., years considered, language, publication status) used as criteria for eligibility, giving rationale. | 5 |
| Information sources | 7 | Describe all information sources (e.g., databases with dates of coverage, contact with study authors to identify additional studies) in the search and date last searched. | 5 |
| Search | 8 | Present full electronic search strategy for at least one database, including any limits used, such that it could be repeated. | 5-6 |
| Study selection | 9 | State the process for selecting studies (i.e., screening, eligibility, included in systematic review, and, if applicable, included in the meta-analysis). | 5-7, Fig.1 |
| Data collection process | 10 | Describe method of data extraction from reports (e.g., piloted forms, independently, in duplicate) and any processes for obtaining and confirming data from investigators. | 5-7 |
| Data items | 11 | List and define all variables for which data were sought (e.g., PICOS, funding sources) and any assumptions and simplifications made. | Table 1- 2 |
| Risk of bias in individual studies | 12 | Describe methods used for assessing risk of bias of individual studies (including specification of whether this was done at the study or outcome level), and how this information is to be used in any data synthesis. | 6-7 |
| Summary measures | 13 | State the principal summary measures (e.g., risk ratio, difference in means). | 6-7, Table 2 |
| Synthesis of results | 14 | Describe the methods of handling data and combining results of studies, if done, including measures of consistency (e.g., I^2^) for each meta-analysis. | 6-7, Table 2 |
| Risk of bias across studies | 15 | Specify any assessment of risk of bias that may affect the cumulative evidence (e.g., publication bias, selective reporting within studies). | 6-7 |
| Additional analyses | 16 | Describe methods of additional analyses (e.g., sensitivity or subgroup analyses, meta-regression), if done, indicating which were pre-specified. | 6-7 |

| **Section/topic** | **#** | **Checklist item** | **Reported on page #** |
| --- | --- | --- | --- |
| **RESULTS** | | |  |
| Study selection | 17 | Give numbers of studies screened, assessed for eligibility, and included in the review, with reasons for exclusions at each stage, ideally with a flow diagram. | 8-10 |
| Study characteristics | 18 | For each study, present characteristics for which data were extracted (e.g., study size, PICOS, follow-up period) and provide the citations. | 8-10, Table 1 |
| Risk of bias within studies | 19 | Present data on risk of bias of each study and, if available, any outcome level assessment (see item 12). | 10, Fig.5 |
| Results of individual studies | 20 | For all outcomes considered (benefits or harms), present, for each study: (a) simple summary data for each intervention group (b) effect estimates and confidence intervals, ideally with a forest plot. | Figures 2-5 |
| Synthesis of results | 21 | Present results of each meta-analysis done, including confidence intervals and measures of consistency. | Table 2,3 |
| Risk of bias across studies | 22 | Present results of any assessment of risk of bias across studies (see Item 15). | 10 |
| Additional analysis | 23 | Give results of additional analyses, if done (e.g., sensitivity or subgroup analyses, meta-regression [see Item 16]). | 6-8 |
| **DISCUSSION** | | |  |
| Summary of evidence | 24 | Summarize the main findings including the strength of evidence for each main outcome; consider their relevance to key groups (e.g., healthcare providers, users, and policy makers). | 11-12 |
| Limitations | 25 | Discuss limitations at study and outcome level (e.g., risk of bias), and at review-level (e.g., incomplete retrieval of identified research, reporting bias). | 12 |
| Conclusions | 26 | Provide a general interpretation of the results in the context of other evidence, and implications for future research. | 13 |
| **FUNDING** | | |  |
| Funding | 27 | Describe sources of funding for the systematic review and other support (e.g., supply of data); role of funders for the systematic review. | 2 |

PLoS Medicine (OPEN ACCESS) Moher D, Liberati A, Tetzlaff J, Altman DG, The PRISMA Group (2009). Preferred Reporting Items for Systematic Reviews and Meta-Analyses: The PRISMA Statement. PLoS Med 6(7): e1000097. doi:10.1371/journal.pmed1000097

**Supplementary Table 2.** The Newcastle-Ottawa Scale (NOS)

| **Author, year** | **Selection** | | | | **Comparability** | | **Exposure** |  |  | **Total** |
| --- | --- | --- | --- | --- | --- | --- | --- | --- | --- | --- |
|  | **S1** | **S2** | **S3** | **S4** | **C1** | **C2** | **E1** | **E2** | **E3** |  |
| Jin et al., 2013 | * | - | * | * | * | * | * | * | - | 7 |
| Zhu et al, 2010 | * | * | * | * | * | * | * | * | - | 8 |
| De Leeuw et al.,2006 | * | * | * | * | * | * | * | * | - | 8 |
| Ribbens et al., 2006 | * | - | * | * | * | - | * | * | - | 6 |
| Zucker et al., 1999 | * | * | * | * | * | * | * | * | - | 8 |
| Ichikawa et al., 1998 | * | * | * | * | * | - | * | * | - | 7 |
| Kotajima et al., 1998 | * | * | * | * | * | - | * | * | - | 7 |
| Akiyama et al., 1997 | * | - | * | * | * | - | * | * | - | 6 |
| Shingu et al., 1995 | * | * | * | * | * | * | * | * | - | 8 |
| Zucker et al., 1994 | * | - | * | * | * | - | * | * | - | 6 |
| Gheita et al., 2015 | * | - | * | * | * | - | * | * | - | 6 |

**Supplementary Table 3.** Comparison of investigated biomarkers in pediatric SLE patients and controls (KPS data).

| *Biomarkers* | **SLE (mean±SD)** | **Control (mean±SD)** | *P* |
| --- | --- | --- | --- |
| **MMP-3** |  |  |  |
| SLE vs. healthy controls | 195.3 ± 15.1 (ng/ml) | 26.4 ± 6.4(ng/ml) | <0.0001 |
| SLE with nephritis vs. without | 211.8 ± 20.0(ng/ml) | 175.4 ± 22.0(ng/ml) | 0.361 |
| SLE with dsDNA Ab vs. without | 177.3 ± 17.7(ng/ml) | 216.8 ± 24.1(ng/ml) | 0.144 |
| **C3** |  |  |  |
| SLE with dsDNA Ab vs. without | 57.4 ± 11.3 (mg/dl) | 95.1 ± 16.2(mg/dl) | 0.068 |
| **C4** |  |  |  |
| SLE with dsDNA Ab vs. without | 8.0 ± 2.9 (mg/dl) | 20.5 ± 4.0(mg/dl) | 0.028 |

* Abbreviations used: dsDNA AB (double-stranded DNA antibodies), MMP-3 (matrix metalloproteinase-3), SLE (systemic lupus erythematosus), C3 (complement 3), C4 (complement 4)

* P values were all two-tailed

**Supplementary Data on National Pediatric SLE group (KPS)**

**Materials and Methods**

*Patients and medical records review*

Patients who were under age 18 at onset, diagnosed as SLE, and visited the department of pediatrics of Severance Children’s hospital or Seoul National University Children’s hospital were recruited. Informed consents were obtained from the parent(s) of the patients for both study participation and publication. SLE was diagnosed according to the criteria by the Systemic Lupus International Collaborating Clinics Classification Criteria (SLICC)[1]. Organ involvement of SLE with respect to the kidneys, joint and nervous system was defined as follows; 1) kidney: proteinuria >0.5 g/24 hours, presence of cellular casts, hematuria with >10 red blood cells/high power field (HPF) excluding infection or stone, >5 leukocytes/HPF excluding infection, or plasma creatinine >1.4 mg%; 2) joints: non-erosive arthritis affecting ≥2 peripheral joints, and 3) nervous system: psychosis, seizure, depression and peripheral neuropathy. Laboratory records were reviewed for abnormalities, including; leukopenia (white blood cell count <4,000/mm^3^), thrombocytopenia (platelet count <100,000/mm^3^), elevated erythrocyte sedimentation rate (>20 mm/hour), the presence of antibodies (dsDNA Ab, antinuclear, anti-Sm), immunoglobulins (Ig G, IgA and IgM, serum levels of complement C3 and C4, and 24-hour-urinary protein excretion (by immunoturbidometry).

*Assays for MMPs*

Venous blood samples were collected into pyogen-free blood collection tubes. Serum was stored at –70°C. Serum levels of MMP-3 were examined by using a commercially available enzyme-linked immunosorbent assay (ELISA) kit (Ab Frontier, Seoul, Korea). The assays were performed according to the manufacturer’s manual. The detection limits of the assays were less than 10 pg/mL. Samples were tested in duplicates.

*Statistical analysis*

The comparison of data between patients was performed by Mann-Whitney U test and Spearman correlation analysis with SPSS version 22.0 software (SPSS, Chicago, Illinois, USA). Each value was presented as the mean ± standard deviation (SD) and *P* values of less than 0.05 were regarded as significant.

*Ethics statement*

The Institutional Review Board and Research Ethics Committee of Yonsei University Severance Hospital and Seoul National University Children’s Hospital approved this study. Our study was conducted according to the ethical standards laid down in the 1964 Declaration of Helsinki and its later amendments.

**Results**

We were able to recruit 11 patients (mean age, 14.5 years; range, 11.8-18 years; 8 females and 3 males) with SLE and 9 healthy controls (mean age, 12.2 years; range, 10-15 years; 7 females and 2 males) with informed consents. The results of the case-control study are summarized in Supplemental Table S3. Between the 11 patients and 9 controls, there were no differences in total white blood cell (WBC) counts, blood urea nitrogen (BUN) and serum cholesterol levels. Serum albumin was significantly lower in the SLE group compared to the control group (*P* = 0.01).

Serum MMP-3 levels were significantly higher in the SLE group than in the control group (195.3 ± 15.1 vs. 26.4 ± 6.4 ng/mL, *P* < 0.001). However, serum MMP-3 levels did not differ between patients with vs. without nephritis or presence of elevated anti-dsDNA titer (Supplementary Table S3).

**References**

1. Petri, M., et al., *Derivation and validation of the Systemic Lupus International Collaborating Clinics classification criteria for systemic lupus erythematosus.* Arthritis Rheum, 2012. **64**(8): p. 2677-86.
